# Upscaling the pharmacy profession in Lebanon: workforce distribution and key improvement opportunities

**DOI:** 10.1186/s12960-019-0386-0

**Published:** 2019-06-24

**Authors:** Mohamad Alameddine, Karen Bou Karroum, Mohamad Ali Hijazi

**Affiliations:** 1 Department of Health Management and Policy, Faculty of Health Sciences, American University of Beirut, Riad El Solh, Beirut, 1107 2020 Lebanon; 2 Department of Pharmaceutical Sciences, Faculty of Pharmacy, Beirut Arab University, P.O. Box: 11 5020, Beirut, Lebanon

**Keywords:** Pharmacist, Workforce planning, Supply, Distribution, Lebanon

## Abstract

**Background:**

The critical role pharmacists play in the healthcare system necessitates close monitoring of their supply, distribution, and competencies. This is especially relevant considering the global increase in the demand for pharmacists. In Lebanon, the pharmacy profession is facing several challenges related to the unplanned supply of pharmacists. The aim of the study is to analyze the Order of Pharmacists registration database and generate workforce distribution over the last six decades.

**Methods:**

This study entailed a detailed database analysis of a deidentified subset of the Order of Pharmacists in Lebanon registration records for years 1954–2018. The analysis generated workforce distribution in terms of growth in number, distribution by gender, age distribution, geographic location, workforce status, sector of employment, and employment position. A descriptive analysis was carried out with cross-tabulations to identify distribution across selected variables.

**Results:**

The pharmacy profession in Lebanon witnessed significant growth over the period of analysis. The “Community” was the most common employment sector (63%), followed by “Pharmaceutical Sector” (24%) and “Hospitals” (5%). The high ratio of pharmacists to 10 000 population (20.3) was contrasted by a low ratio of hospital pharmacist per hospital and per 100 hospital beds (1.9 and 1.7, respectively).

The high pharmacist to population ratio is indicative of an oversupply of pharmacists precipitated by poor workforce planning. While oversupply did not result in high unemployment, it may have negatively affected the income of pharmacists and their adherence to the professional and ethical requirements of their job. Despite the large number of pharmacists in Lebanon, findings elicit concerns over the adequacy and safety of pharmacists’ services in hospitals with a low ratio of hospital pharmacists to 100 hospital beds.

**Conclusion:**

This study unearthed several opportunities and concerns related to planning the supply, distribution, regulation, and the professional scope of practice of pharmacists in Lebanon. A concerted multi-stakeholder effort to address some of the identified challenges will not only improve the professional status of pharmacists but will also enhance the safety and efficiency of pharmaceutical care in the country.

## Background

Pharmacists are the healthcare professionals responsible for ensuring optimal medication therapy outcomes to all citizens at the community and institutional levels [1]. The World Health Organization (WHO) introduced an expanded scope of practice of pharmacists who are expected to function as caregivers, decision-makers, communicators, managers, life-long learners, teachers, leaders, and researchers [2]. In some countries, such expanded roles include pharmacy-based preventative care counseling, including blood glucose level, blood pressure, cholesterol, and nutrition and vaccination counseling. Pharmacists are further expected to work effectively within a multi-disciplinary team of healthcare providers and are required to provide patient-centered pharmaceutical services at the patients’ bedside [3].

In the past few decades, significant challenges have occurred within the profession of pharmacy leading to the enhancement of the patient-centered clinical role of pharmacists. Clinical pharmacy interventions are not only beneficial to save millions of lives [4], but were also proven to generate good cost savings a positive return on investment [5]. The clinical pharmacy roles, responsibilities, and services are well introduced in the healthcare systems of developed countries yet are regretfully not well articulated in many developing contexts [6, 7].

The critical role that pharmacists play in the healthcare system necessitates close monitoring of their supply, distribution, and competencies. This is especially relevant in light of the global demographic and epidemiological transition which increased the demand on all healthcare services in general and the services of pharmacists in particular [8]. The imbalance between the demand and the supply of pharmacists varies between countries and is directly related to the expenditure on health and economic development. The maldistribution of pharmacists between rural and urban areas and the public and private sectors might lead to shortage in certain regions [9].

In the Middle East Region, there is a dearth of systematic examinations of the pharmacist workforce trends and distribution, as well as the adequacy of supply to meet population needs in published literature. Having said that, there have been previous attempts to investigate the pharmacy workforce, yet such studies majorly focused on measuring the challenges that pharmacists face or assessing the quality of their practice environment. For example, the Jordanian pharmacists’ level of stress and dissatisfaction were examined and were found to be associated with poor work relationships with physicians, patient workload, long work hours, and lack of professional advancement [10]. Additional challenges facing Jordanian pharmacists included the accreditation of pharmacy education, requirements for pre-registration, workforce planning and development, and wage rate [11]. Obstacles facing pharmacists were also investigated in the United Arab Emirates (UAE) and included lack of physicians’ recognition and shortage in patient demand for pharmaceutical services [12]. Additional challenges facing UAE pharmacists included the high turnover rate of community pharmacists, the selling of medications without a prescription, and a deficiency in accessing up-to-date sources of information [12].

In Lebanon, registration with the Order of Pharmacists (OPL) is a prerequisite for practicing the profession of pharmacy. At the time of this analysis, OPL records indicated a total number of 8855 registered pharmacists and 3198 licensed pharmacies serving an estimated population of around four million individuals. Pharmacy students can graduate with a bachelor’s in pharmacy degree (5-year pharmacy program) or a PharmD degree (6-year pharmacy program), with the possibility of higher education to obtain a masters or a Doctor of Philosophy (PhD) degree. Community pharmacies are privately owned on a for-profit basis and are the only legal provider of prescription and non-prescription drugs to the Lebanese community. According to the OPL and the Ministry of Public Health (MOPH) laws, MOPH-licensed and OPL-registered pharmacists should be present at all times during the pharmacy’s opening hours [13].

The pharmacy profession in Lebanon has been facing multiple challenges relating to the practicing of the profession and the protection of the professional status of pharmacists. Previous studies showed that pharmacists in Lebanon are not satisfied with multiple issues including the distribution of pharmacies, drug prices, profit margin, policies governing the profession, prescribing ethics, sale of counterfeit drugs, and political intervention [13–15]. Other studies described the pharmacy practice behavior, attitudes, and knowledge of pharmacists in Lebanon [16–22]. However, a systematic longitudinal investigation of the workforce numbers and demographic characteristics has not been carried out yet nor has there been an examination of the employment status, geographic distribution, or allocation across work sectors. Such investigations are essential to guide current and future workforce planning efforts and to guide pharmacists to serve areas and sectors of need.

### Specific aims

The aim of the study is to analyze a deidentified subset of the OPL registration database and generate workforce distribution in terms of growth in number and distribution of pharmacy professionals in Lebanon. It would help unearth key workforce distribution that could guide education, training, and regulation of pharmacists.

## Methods of inquiry and analysis

Then, the study carried out a detailed quantitative analysis of a deidentified subset of the OPL registration database (1954–2018) to generate the workforce distribution of pharmacists in Lebanon. The database was retrieved and prepared for proper analysis using IBM SPSS. After that, the following variables were included (Table 1).

**Table 1:** The definition of key variables included in this study

Variable	Definition
The annual total number of pharmacists	The total number of pharmacists that are newly registered or had a continued registration with OPL at the end of a registration year
Gender	Total number of females and males in the registration database
Age of pharmacists	The age of the registered pharmacist derived from their date of birth
Workforce status	Active: A pharmacist registered with the OPL who reported actively working as a pharmacist in Lebanon in the last cycle of registration renewal. Unemployed: A pharmacist registered with the OPL who reported unemployment in the last cycle of registration renewal Working outside the country: A pharmacist registered with the OPL who reporting working outside Lebanon in the most recent cycle of registration renewal.
Work governorate	The Governorate where the Pharmacist works (Beirut, Mount Lebanon, North Lebanon, South Lebanon, Beqaa, and Nabatieh)
Sector of employment	Grouped into the following categories: Community: Pharmacist providing service in community pharmacies as employers or owners Hospital: Pharmacist employed in hospitals as hospital or clinical pharmacists (whether in the pharmacy department or at the floor) Pharmaceutical sector: Pharmacists employed in the pharmaceutical companies in sales, marketing, policy, research or development positions Academic sector: Pharmacist employed in academic institutions Others: Pharmacists working in any other sector
Graduation university location	Divided into the following: Local (completed the pharmacy degree in Lebanon) versus Overseas (completed the pharmacy degree outside Lebanon)
Employment position	The job position reported upon registration (for new registrants) or during the most recent renewal of registration process (for previously registered pharmacists)

We carried out a descriptive analysis on all study variables with some cross-tabulations to identify distribution across some key variables. The pharmacist to population ratio and pharmacist to bed ratio were calculated in light of the updated number of hospitals and beds acquired from the Syndicate of Hospitals in Lebanon. The study analyzed the following indicators (Table 2).

**Table 2:** Definition of key indicators used in this study along with their source

Key Indicator	Numerator (source)	Denominator (source)
Pharmacists to population ratio (per 10 000)	Total number of pharmacists registered with OPL (OPL database)	Total number of population in Lebanon (according to the latest census)
Pharmacies to population ratio (per 10 000)	Total number of licensed pharmacies in Lebanon (OPL database)	Total number of population in Lebanon (according to the latest census)
Ratio of community pharmacists to pharmacies	Total number of community pharmacists in Lebanon (OPL database)	Total number of licensed pharmacies in Lebanon (OPL database)
Ratio of hospital pharmacists to hospitals	Total number of hospital pharmacists in Lebanon (OPL database)	Total number of hospitals in Lebanon (acquired from the syndicate of hospitals)
Ratio of hospital pharmacists to hospital beds (per 100)	Total number of hospital pharmacists in Lebanon (OPL database)	Total number of hospital beds in Lebanon (acquired from the syndicate of hospitals)

## Results

Analysis started with examining selected sociodemographic and work-related characteristics using the OPL database. Table 3 reveals that the total number of pharmacists in Lebanon is 8855 with females constituting the majority (62.3%). The workforce is relatively young with 56.2% of Lebanese pharmacists aged 39 years or less and 43% registered with OPL for 9 years or less. The vast majority of Lebanese pharmacists are actively working (91.3%) with a small proportion working outside Lebanon (8.1%) or unemployed (0.6%).

**Table 3:** Socio-demographic and work-related characteristics of pharmacists from the registration database of OPL

Variable	Total sample (*n* = 8 855) *N* (%)
Age (years)	
≤ 30	1574 (17.8)
30–39	3400 (38.4)
40–49	2059 (23.3)
≥ 50	1822 (20.6)
Gender	
Males	3337 (37.7)
Females	5518 (62.3)
Location of university of graduation	
Inside Lebanon	6280 (69.9)
Outside Lebanon	2575 (29.1)
Employment status	
Working	8078 (91.3)
Abroad	720 (8.1)
Unemployed	57 (0.6)
Years of registration with OPL (experience)	
1–9	3807 (43.0)
10–19	2631 (29.7)
20–29	1781 (20.1)
30–39	415 (4.7)
≥ 40	221 (2.5)
Sector of employment	
Community	4113 (63.4)
Hospital	328 (5)
Pharmaceutical	1557 (24)
Academic	248 (3.8)
Others	240 (3.7)

Regarding the employment sectors, the “Community” was the most common employment sector (63%), followed by “Pharmaceutical Sector” (24%) and “Hospitals” (5%). Most of the pharmacists in the registration database graduated from universities located inside Lebanon (70%).

We next examined the absolute and relative distribution of pharmacists and pharmacies in Lebanon by governorate. Table 4 reveals that close to half of Lebanese pharmacists (48.5%) live in Mount Lebanon, followed by the capital, Beirut (17.4%) and North Lebanon (11.4%).

**Table 4:** Proportion of pharmacists and pharmacies relative to population by Governorate [23]

Governorate/distribution	Pharmacist		Population		Pharmacy		Pharmacist to population ratio (per 10 000)	Pharmacy to population ratio (per 10 000)
	No.	%	No.	%	No.	%		
Beirut	1537	17.4	407 453	9.35	235	7.3	37.7	5.8
Beqaa	876	9.9	620 963	14.26	507	15.9	14.1	8.2
Mount Lebanon	4297	48.5	1 550 745	35.60	1 366	42.7	27.7	8.8
Nabatieh	387	4.4	329 803	7.57	242	7.6	11.7	7.3
North	1011	11.4	938 955	21.56	471	14.7	10.8	5.0
South Lebanon	746	8.4	507 995	11.66	377	11.8	14.7	7.4
Total	8855	100	4 355 913	100.00	3 198	100	20.3	7.3

As for the pharmacist to population ratio, Table 4 reveals that the average ratio of pharmacist to population was 20.3 per 10 000 population. The highest ratio was in Beirut (37.7) followed by Mount Lebanon (27.7). In contrast, the lowest ratio was in Nabatieh and North Lebanon (11.7 and 10.7, respectively).

With respect to pharmacies, Table 4 exhibits that Mount Lebanon and Beqaa Governorates had the highest ratio of pharmacies to 10 000 population (8.8 and 8.2, respectively). Interestingly, although the Beirut Governorate had the highest pharmacist to population ratio nationally, it had the second lowest pharmacy to population ratio, with 5.8 pharmacies per 10 000 population. The North Lebanon Governorate had the lowest pharmacist to population ratio (5 pharmacies/10 000 population). The average national pharmacy to population ratio was 7.3 per 10 000 population.

Table 5 provides a more detailed analysis of the number and proportional distribution of community pharmacists in Lebanon. The table reveals that the Mount Lebanon Governorate hosts 43.9% and 42.7% of community pharmacists and pharmacies, respectively. Furthermore, the average ratio of community pharmacists to pharmacies in Lebanon is about 1.3. This ratio is comparable among all governorate ranging from 1.1 to 1.3, except for the governorate of Beirut where the ratio is 2.1 community pharmacists per pharmacy.

**Table 5:** Proportional distribution of community pharmacists to pharmacies by Lebanese Governorate in 2018

Governorate	Number of community pharmacists	Proportional distribution of community pharmacists	Number of pharmacies	Community pharmacist/pharmacies
Beirut	496	12.1	235	*2.1*
Beqaa	539	13.1	507	*1.1*
Mount Lebanon	1804	43.9	1366	*1.3*
Nabatieh	276	6.7	242	*1.1*
North	551	13.4	471	*1.2*
South Lebanon	446	10.8	377	*1.2*
Total	4112	100.0	3198	*1.3*

Contrary to the large number of community pharmacists, Lebanon has a relatively modest number of pharmacists working in hospitals (total = 328, Table 6). Note that for the sake of this analysis and to match the reporting style of the Syndicate of Hospitals in Lebanon, the Nabatieh Governorate (located in Southern area of Lebanon) was merged with that of South Lebanon. If we examine the ratio of hospital pharmacists employed to hospitals, we realize that the national average is 1.9 pharmacists per hospital and 1.7 pharmacists per 100 beds. There is also a clear national discrepancy in the distribution of hospital pharmacists. Again, Mount Lebanon has the highest ratio of hospital pharmacists per hospitals and per 100 beds (2.6 and 3.2, respectively) followed by Beirut (1.7 and 2.8, respectively). In the South, although the ratio of hospital pharmacists per hospital is 1.7, the governorate has the lowest ratio per 100 beds (1.1). In contrast, the North Governorate shows the lowest ratio per hospital (1.2) but a higher ratio per 100 beds (2.1) as compared to the average national ratio (1.7).

**Table 6:** Ratio of hospital pharmacists to hospitals and beds by governorate 2018

Governorate	Number of hospitals	Hospital pharmacists	Number of beds	Hospital pharmacists/hospitals	Hospital pharmacist/beds (100)
Beirut	34	57	2 783	*1.7*	*2.8*
Beqaa	24	37	2 107	*1.5*	*2.1*
Mount Lebanon	59	151	6 153	*2.6*	*3.2*
North	30	37	2 309	*1.2*	*2.1*
South Lebanon (Nabatieh and South)	27	46	5 476	*1.7*	*1.1*
Total	*174*	*328*	*18 828*	*1.9*	*1.7*

**Fig. 1:**
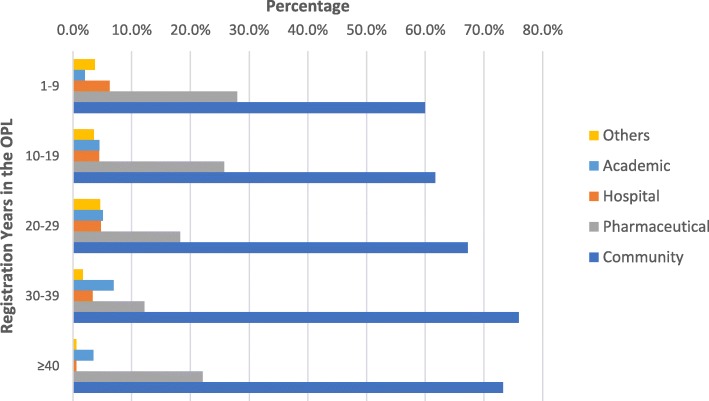
Distribution of pharmacists by the number of registration years and sector of employment

We finally examined the distribution of pharmacists in Lebanon by their years of experience and sector of employment. Figure 1 reveals that the dominant employment sector of pharmacists in Lebanon among all age groups is the Community sector (from 60 to 76%) followed by “Pharmaceutical” (from 12 to 28%). Interestingly, the figure shows also that newly registered pharmacists are relatively higher represented in the pharmaceutical sector. As their experiences grow up, they become more represented in the community sector. In addition, the proportion of pharmacists reporting employment in academia grows with more experienced pharmacists before falling again for the more than 40 years of age group. In contrast, the employment in the hospital sector relatively decreases with age.

## Discussion

Our analyses reveal that Lebanon hosts 8855 pharmacists with a female majority (62.3%). Most Lebanese pharmacists are indeed working as community pharmacists (63%). This is in good agreement with the global workforce distribution of pharmacists, where the majority of pharmacists are employed in the community sector [24]. Female pharmacists represent a higher percentage in the European and Africa/Eastern Mediterranean region, where a higher percentage of male pharmacists appear in the Western Pacific/ South East Asia region [8].

The data showed that Lebanon has a pharmacist to population ratio of 20.3 pharmacists per 10 000 population. This is quite high compared with the 2017 median global density of pharmacists to population (5.09 pharmacists per 10 000 population) [25]. It is even higher than the average pharmacist to population ratio reported by OECD Health report in 2017 (8.2 pharmacists per 10 000 population) [24]. Lebanon not only has a ratio that is double that of the average ratio of the OECD countries, but also a ratio higher than that of Japan, which is the highest among OECD countries (17 pharmacists/10 000 population). Other developed countries report lower ratios, for example, France (11.2), the United States of America (9.2), Canada (10.4), the United Kingdom (8.3), and Germany (6.4) [24].

The high pharmacist to population ratio is indicative of poor workforce planning and a general lack of regulation to the pharmacy education sector. It may also be indicative of a potential oversupply of pharmacists. The experience of other countries shows that this may enhance the risk of unemployment among pharmacy graduates or it may force pharmacists’ income down [26]. However, unemployment among pharmacy graduates is not the case in Lebanon since only a minute proportion of pharmacists (0.6%) report unemployment. This could be explained by the following: 1—the distribution of pharmacists across various sectors of employment with a good proportion working in the pharmaceutical sector, 2—the large number of community pharmacies in Lebanon (one pharmacist per pharmacy), or 3—the proportion of pharmacists who may be keeping their registration active and indicating employment to benefit from the retirement benefits of the OPL. While unemployment does not surface as an issue, reduced income and profit margin certainly do [13–15]. Future studies are recommended to validate the explanations offered above and better guide policymaking concerning pharmacy workforce planning.

Examining the ratio of pharmacies to population reveals a concern with the inequitable distribution of pharmacies taking into consideration the large difference in the ratio of pharmacies among governorates. These variations can be explained by the conditions necessary to license the opening of a new pharmacy. According to OPL Decree No. 2622 issued in 1992, a distance of 300 m (measured as the shortest distance that can be reached) is required between new and the closest existing pharmacy regardless the population density. The average population served by a pharmacy could be used as an indication of country’s community pharmacy infrastructure and capacity, as well as the accessibility of pharmacy services and, to some extent access to medicine. At the global level, the median stands at 2.4 community pharmacy per 10 000 inhabitants [25]. Compared with OECD countries, the ratio of pharmacies in Lebanon (7.3/10 000 population) is three times higher than that of the average ratio of OECD (2.4 pharmacies/10 000 population) and is higher than the highest ratio reported to Spain (4.7) [27]. Other countries with leading health systems report much lower ratios like France (3.4), Canada (2.6), Germany (2.4), and the United Kingdom (2.2) [27].

These relatively high numbers of pharmacists and pharmacies in Lebanon are negatively influencing the professional status and income of pharmacists and are leading to a general dissatisfaction among pharmacists [15]. Perhaps more disconcerting, this oversupply might lead to illegal or unethical competition in pharmacy practice with concerns including price discounts/illegal offers, indirect promotions, dispensing without prescriptions, and saving costs by employing non-pharmacists to dispense drugs in community pharmacies.

A recent FIP report recommends that countries proactively model their future workforce needs and develop planning strategies to secure the future provision of services and education within the profession [26]. Managing the oversupply of health professionals entails key decision-makers’ employing selected policy options to regain the balance between workforce supply and demand. Such options include the following: 1—decreasing the number of pharmacy graduates by enforcing maximum acceptance quotas on pharmacy schools and restricting licensure to new ones, 2—restricting entry for students studying outside Lebanon, 3—encouraging the sharing of pharmacists with other countries, or 4—changing policies to enhance employment of pharmacists (e.g. mandating a minimum number of pharmacists per a certain number of hospital beds).

The ratio of community pharmacists to pharmacies (1.3) indicates that most community pharmacists are served by one pharmacist who is most likely the owner of the pharmacy. The global median of pharmacists per community pharmacy stands at 1.67 and the vast majority of countries have regulations that require the presence of a pharmacist at community pharmacies whenever they are open to the public [25]. The same regulations are applied in Lebanon, where a pharmacist can only incorporate his or her license into one drugstore or drug company, and pharmacies can only be owned by a pharmacist registered with OPL. Chain pharmacies are prohibited. If the pharmacy opens 24 h, the license of two pharmacists should be incorporated [28]. The low ratio of community pharmacists to pharmacies in Lebanon (1.3) raises a serious public health concern since the opening hours of pharmacies in Lebanon ranges from minimum 8–10 h to max 24 h. It is logistically very difficult for one pharmacist to ensure continuous presence at the pharmacy to exercise the role of patient advocate and experienced pharmaceutical care provider as stipulated by the law. Thus, the findings cast double whether we have the continuous presence of pharmacists in the pharmacy (note Lebanon does not have assistant pharmacist degrees) and how the pharmacist is managing their workload without affecting the quality of their patient-care services and safety of the population.

Community pharmacists in Lebanon are struggling to strike the balance between the business and professional missions. The community pharmacist in Lebanon is a healthcare professional who is primarily reimbursed through the sale of products rather than through the provision of services. Nevertheless, because drugs continue to grow in potency risk and cost, patients are going to need more personal care. Pharmacists are becoming personally responsible for educating their patients on the use of both prescription and over-the-counter medications. Unfortunately, if pharmacies remain improperly staffed, a burdensome workload will dilute the professional roles of pharmacists and may jeopardize the safe consumption of drugs [14]. Policy and decision-makers are urged to reflect on the findings to decipher evidence-based recommendation that would safeguard the interests of the consumer and the pharmacist alike. Such recommendations may include the following: 1—organizing the opening hours of pharmacies based on the number of employed pharmacists, 2—advocating for higher salaries and benefits for employed pharmacists to decrease the drive to open own pharmacy, or 3—expanding the professional scope of practice of the pharmacies to include counseling services and medication management rather being restricted to products sales only.

Lebanon has a modest number of hospital pharmacists (328–5%). The average ratio of hospital pharmacists to hospitals and to 100 hospitals beds was 1.9 and 1.7, respectively (Table 6). The ratios varied significantly among governorates, which is indicative of a lack of proper regulation of the number of pharmacists in hospitals or per beds to ensure safe and effective patient care. Beyond numbers, and perhaps more disconcerting, is the fact that hospital pharmacists in Lebanon are still in their dispensary-based role rather than the clinical or patient care role (although few medical centers in Lebanon started to employ clinical pharmacists). The hospital pharmacist’s role in Lebanon focuses on the distribution of medications, compounding, formulary review, and cost management [28]. The scope of practice of hospital pharmacists is much wider as it includes ensuring safe, appropriate, efficacious, judicious, and cost-effective use of medications while decreasing improper medication usage and enhancing the satisfaction of patients [29, 30]. Such professional role is far from being achieved with the shy numbers of hospital pharmacists in Lebanon. The country thus has the opportunity to direct the oversupply of community pharmacists toward clinical pharmacy and benefit from the availability of PharmD programs in local universities. The pharmacist role in the hospital setting should be expanded from a majorly dispensary-based role to a more clinically focused one based on the ward. Some countries in the region are moving in this direction. For example, Saudi Arabia released new Guidelines on Hospital Pharmacy Manpower to achieve a ratio of 20 pharmacists per 100 beds in 2030 [31].

However, upscaling the supply of clinical pharmacists in Lebanon is not without challenges, including the lack of support from physicians and the deficiency in the necessary regulation and health policies. Identifying and addressing such obstacles is a prerequisite to enhancing the role of clinical pharmacists in the country.

Some shortcomings in this study are worth reporting: First, despite the best efforts of OPL, it cannot be ascertained that all the records in the Order of Pharmacists database are updated. Second, the numbers of active pharmacists in Lebanon may be inflated since a proportion of pharmacists will keep their registration active to benefits from the registration and retirement benefits of OPL. Third, the generated pharmacists to population ratios may not be accurate since there is no recent census of the number of individuals living in Lebanon and population estimates may not account for the large number of refugees residing in the country. Last, the fact that a pharmacist reports working in a particular sector does not ascertain that the pharmacist’s actual scope of practice is appropriate for that sector. For example, most hospital pharmacists are not providing clinical pharmaceutical services.

This study offered a detailed and benchmarked systematic examination of the numbers and distribution of pharmacists in Lebanon. It unearthed several opportunities and concerns related to regulating the supply, distribution, regulation and the professional scope of practice of pharmacists in Lebanon. Upscaling the pharmacy profession necessitates concerted and coordinated efforts by all concerned stakeholders to address the identified challenges. This will not only improve the professional status of pharmacists in Lebanon but will also enhance the safety and efficiency of access to pharmaceutical services in the country.

## Data Availability

All data used in this study are available at the Lebanese Order of Pharmacists.

## Abbreviations

FIP: International Pharmaceutical Federation
MoPH: Ministry of Public Health
OECD: Organization for Economic Co-operation and Development
OPL: Order of Pharmacists in Lebanon
PhD: Doctor of Philosophy
UAE: United Arab Emirates
WHO: World Health Organization
